# Supplementary material for: WordCommentsAnalyzer: A windows software tool for qualitative research
Source: F1000Res. 2018 Sep 4;7:536. Originally published 2018 May 3. [Version 2] doi: 10.12688/f1000research.14819.2 (PMC6137411; doi:10.12688/f1000research.14819.2)
Supplement: Supplementary file 3 [file f1000research-7-17614-s0002.tgz › b2a79496-4ada-47c4-9682-a99ce867836d.zip!/Eight Iranian Health Journals/Salmand_Iranian Journal of Ageing _16.docx]

© Salmand_Iranian Journal of Ageing . This work is licensed under CC BY-NC 4.0 (https://creativecommons.org/licenses/by-nc/4.0/).

Volume & Issue: دوره 9 - شماره 2

Title: ارتباط بین بیماری‌های مزمن و ناتوانی در انجام فعالیت‌های روزانه زندگی، در سالمندان شهر امیرکلا

http://salmandj.uswr.ac.ir/article-1-601-fa.html

اهداف: با افزایش سن، بر تعداد بیماری‌های مزمن افزوده می‌شود. این بیماری‌ها ممکن است منجر به مرگ فرد نشوند، اما کیفیت زندگی شخص مبتلا را به خطر می‌اندازند و می توانند ناتوانی‌های زودرس و شدید ایجاد کنند. هدف از این مطالعه تعیین ارتباط بین این بیماری‌ها و ناتوانی در انجام فعالیت‌های روزانه زندگی سالمندان شهر امیرکلاست.  مواد و روش‌ها: این مطالعه توصیفی-تحلیلی مقطعی بر روی سالمندان شهر امیرکلا انجام شد. اطلاعات با استفاده از پرسش‌نامه‌ای که شامل مشخصات دموگرافیک و پرسش‌هایی در مورد ناتوانی بود (معیار Katz) اندازه‌گیری شد. سپس داده‌ها توسط نرم‌افزار SPSS 15 و آزمون‌های آماری رگرسیون لجستیک، تی و مجذور کای تجزیه و تحلیل گردید. مقدار P کمتر از 0/05 به‌عنوان سطح معنی‌دار درنظرگرفته شد. یافته‌ها: در این بررسی 1017 سالمند مورد مطالعه قرار گرفتند. %85 افراد در انجام کلیه فعالیت های روزانه زندگی مستقل و %15 حداقل در یک مورد وابستگی داشتند. سکته مغزی (001/OR=7/2, 95%CI=3/97, P<0)، سرطان (OR=6/80, 95%CI=2/53-18/26, P<0/001) و کاهش شنوایی (001/OR=4/58, 95%CI=3/01-6/98, P<0) بیشترین نقش را در ایجاد ناتوانی در سالمندان داشتند. در این بررسی، زنان در کلیه فعالیت‌های روزانه زندگی ناتوان‌تر از مردان بودند به‌طوری‌که سکته مغزی (001/OR=10/12, 95%CI=4/06-25/2, P<0)، سرطان (001/OR=6/08, 95%CI=1/29-28/6, P<0) و کاهش بینایی (OR=4/26, 95%CI=2/32-7/82, P<0/001) بیشترین ناتوانی را در این گروه ایجاد می‌کرد.  نتیجه‌گیری: این مطالعه نشان داد شیوع بیماری‌های مزمن و ناتوانی در انجام فعالیت‌های روزانه زندگی در زنان بیش از مردان سالمند است و سکته مغزی، سرطان و کاهش شنوایی و بینایی می‌تواند با کاهش عملکرد انجام فعالیت‌های روزانه زندگی سالمندان ارتباط داشته باشد. ازاین‌رو برنامه‌ریزی‌های مناسب برای غربالگری و کاهش عوامل خطر این بیماری‌ها ضروری به‌نظر می‌رسد.

Title: نقص توجه بیماران آلزایمر در اجرای تکلیف دوگانه

http://salmandj.uswr.ac.ir/article-1-620-fa.html

اهداف: هدف پژوهش حاضر، ارزیابی نقص تقسیم توجه در بیماران آلزایمر (AD) با استفاده از روش تکلیف دوگانه است تا مشخص شود که آیا از این روش می‌توان برای تشخیص زودهنگام AD استفاده کرد یا خیر. مواد و روش‌ها: 23 فرد مسن (یازده زن و دوازده مرد) به طور داوطلبانه در تحقیق شرکت کردند: سیزده بیمار AD و ده فرد مسن سالم گروه کنترل. کاربندی آزمایشی شامل کوشش‌های مجزا و هم‌زمان با دو سطح دشواری بود. در وضعیت تک‌کاره از شرکت‌کنندگان خواسته شد ماه‌های سال را با ترتیب معمولی (آسان) و معکوس (دشوار) پشت سرهم بیان کنند. آن‌ها یک تکلیف بینایی، فضایی و حرکتی کامپیوتری از نوع پیگردی را نیز اجرا کردند. در وضعیت دوگانه، شرکت‌کنندگان هم زمان با اجرای تکلیف پیگردی، یکی از تکالیف یادآوری ماه‌های سال را نیز اجرا می‌کردند.  یافته‌ها: یک اثر تعاملی (بیماری×سطح دشواری) معنادار مشاهده شد. به این صورت که در بیماران AD افت عملکرد اجرای هم‌زمان دو تکلیف تابع سطح دشواری تکلیف بود، در حالی که برای افراد مسن گروه کنترل این گونه نبود.  نتیجه‌گیری: نتایج تحقیق حاضر درک ما در زمینه نقص‌های توجه بیماران AD را افزایش می‌دهد و نیز نشان می‌دهد در ارزیابی توجه افراد مبتلا به مشکلات شناختی، باید نقش بار شناختی تکالیف به‌کار رفته در روش تکلیف دوگانه نیز به‌عنوان یک عامل میانجی در نظر گرفته شود.

Title: پاسخ عصبی‌عضلانی بیماران مبتلا به مالتیپل اسکلروزیس به ارتعاش‌درمانی

http://salmandj.uswr.ac.ir/article-1-596-fa.html

اهداف: هدف از این پژوهش، تأثیر ارتعاش کل بدن بر بهبود عملکرد بیماران مبتلا به ام .اس است.  مواد و روش‌ها: در این تحقیق، هفتادوسه نفر از افراد مبتلا به ام. اس با میانگین سنی 2/63±73 سال حضور داشتند و بیست نفر از این افراد با رضایت کامل شرکت کردند. آن‌ها ناتوانی متوسط در دامنه 2/5تا 4/5 داشتند. آزمودنی‌ها براساس طرح بوسکو، در چهار گروه تقسیم شدند: یک گروه کنترل و سه گروه تجربی. گروه اول در شش جلسه و در شش هفته کار خود را انجام دادند و گروه دوم در دوازده جلسه و در شش هفته و گروه آخر نیز در هجده جلسه و در شش هفته. فرکانس ارتعاش ۱۵ تا ۲۵ هرتز استفاده شد. پس از پایان برنامه تمرینی، عملکرد عصبی-عضلانی با آزمون استاندارد chair-5 و TUG ارزیابی شد. به منظور تحلیل داده‌ها، از SPSS نسخه 18 در سطح معناداری آلفای 5 درصد استفاده شد.  یافته‌ها: نتایج روش‌های آماری تحلیل پراکنش با اندازه‌گیری مکرّر و تحلیل پراکنش یک راهه، نشان دادند تمرین ارتعاش کل بدن، زمان انجام آزمون P<0/03) chair-5) و آزمون tug (P<0/03) را به طور معناداری کاهش می‌دهد. برای آزمون chair-5، سه جلسه تمرین در هفته از نوع تمرین ارتعاش کل بدن، به‌طور معنادارتری عملکرد عصبی-عضلانی بهتری در مقایسه با دو گروه دیگر داشت (0/03>P). نتیجه‌گیری: تمرینات ارتعاش کل بدن در بیماران ام .اس به بهبود عملکرد عصبی-عضلانی منجر می‌شود.

Title: تاثیر ورزش و فعالیت بدنی روی زمان واکنش و پاسخ سالمندان

http://salmandj.uswr.ac.ir/article-1-588-fa.html

اهداف: فعالیت‌های بدنی تأثیر زیادی بر ارتقای عملکرد حرکتی و شناختی سالمندان دارد. هدف از انجام این پژوهش، بررسی تأثیر ورزش و فعالیت بدنی بر زمان واکنش و زمان پاسخ سالمندان است.  مواد و روش‌ها: روش پژوهش حاضر، علمی-مقایسه‌ای و جامعه آماری آن شامل سالمندان شهرستان مهاباد است. از میان این جامعه آماری، سی‌نفر غیرفعّال به‌طور تصادفی و سی نفر فعال به‌طور در دسترس انتخاب شدند و زمان واکنش و پاسخ آن‌ها اندازه‌گیری شد. زمان واکنش به وسیله دستگاه زمان سنج واکنش، ساخت شرکت «تاکی»، مدل YB 1000 و زمان پاسخ نیز از طریق آزمون میدانی حرکت و پاسخ انتخابی نلسون اندازه‌گیری شد. ابتدا زمان واکنش و سپس زمان پاسخ اندازه‌گیری شد. برای تحلیل داده‌ها، از آمار توصیفی و آزمون K-S و آزمون تی مستقل استفاده شد.  یافته‌ها: آزمون کلموگروف-اسمیرنوف نشان داد داده‌های تحقیق، توزیعی طبیعی دارند. طبق نتایج این پژوهش، ورزش و فعالیت بدنی تأثیر مثبتی بر زمان واکنش و زمان پاسخ دارد؛ به‌طوری‌که نتایج آزمون تی مستقل نشان داد گروه فعال به طور معناداری، زمان واکنش (P=0/000)  و زمان پاسخ (000/P=0) کوتاه‌تری نسبت به گروه غیرفعال داشتند.  نتیجه‌گیری: نتایج این تحقیق نشان می‌دهد ورزش و فعالیت بدنی از طریق تغییرات مثبت فیزیولوژیکی و روانی-حرکتی، زمان واکنش و زمان پاسخ سالمندان را کاهش می‌دهد.

Title: تاثیر نگهداری از حیوانات خانگی بر افسردگی سالمندان ساکن در آسایشگاه‌های سالمندان

http://salmandj.uswr.ac.ir/article-1-595-fa.html

اهداف: افسردگی از مسائل شایع دوره سالمندی است که میزان آن در سالمندان مقیم آسایشگاه‌های سالمندان، سی تا پنجاه درصد می باشد. روش‌های بسیاری از جمله دارودرمانی برای درمان افسردگی به کار می رود ولی با توجه به اینکه معمولاً سالمندان به علت مشکلات متعدد، داروهای زیادی مصرف می‌کنند، استفاده از روش‌های غیردارویی مانند درمان به کمک حیوانات می‌تواند مورد توجه قرار گیرد. این مطالعه با هدف بررسی تأثیر نگهداری و مراقبت از حیوانات خانگی بر میزان افسردگی سالمندان انجام گردید.  مواد و روش‌ها: این پژوهش از نوع نیمه‌تجربی بوده که در آن شصت نفر سالمند مقیم سراهای سالمندان به عنوان نمونه‌های پژوهش انتخاب و به طور تصادفی در دو گروه مداخله (29 نفر) و گروه شاهد (31 نفر) تخصیص یافتند. هر دو گروه با ابزار گردآوری داده‌ها شامل: پرسش‌نامه اطلاعات دموگرافیک، آزمون افسردگی سالمندان مورد بررسی قرار گرفتند و سپس مداخله پژوهش شامل برنامه نگهداری از حیوانات طی یک دوره شش هفته‌ای در گروه مداخله انجام گردید و در گروه شاهد این مداخله انجام نشد. در پایان دوره مداخله، مجدداً اعضای دو گروه با آزمون افسردگی سالمندان ارزیابی و نتایج حاصل قبل و بعد از مداخله، با یکدیگر مقایسه شدند. برای تجزیه و تحلیل داده ها از آزمون‌های کای اسکوئر و تی مستقل و همچنین برای بررسی متغیرها در بین دو گروه از آزمون های تی مستقل استفاده شد. یافته‌ها: نتایج نشان داد که میانگین افسردگی سالمندان گروه مداخله از 8/55 قبل از مداخله به 4/79 بعد از مداخله کاهش یافت (0/001>P). این در حالی است که در گروه شاهد، قبل و بعد از مداخله تفاوت معنی‌داری مشاهده نشد. مقایسه میانگین نمرات افسردگی در دو گروه پیش از مداخله، تفاوت آماری معناداری را نشان نمی دهد (531/P=0)، درحالی که میانگین نمرات افسردگی پس از مداخله در گروه شاهد به‌طور معناداری بیشتر از گروه مداخله است (001/P=0) نتیجه‌گیری: یافته‌های پژوهش حاضر ضمن حمایت از فرضیه‌های تحقیق نشان داد که این مداخله می‌تواند سبب بهبود محسوس و معنادار افسردگی سالمندان شود؛ لذا انجام مداخلات غیرتهاجمی نظیر نگهداری از حیوانات توسط پرستاران می‌تواند ضمن بهبود افسردگی سالمندان زمینه را جهت حضور فعال‌تر آن‌ها در محیط فراهم کند. بنابراین به‌کارگیری گسترده‌تر این نوع مداخله در آسایشگاه‌های سالمندان توصیه می‌شود. ضمناً این مطالعه یک حوزه جدید جهت پژوهش‌ها و خدمات پرستاری ایجاد کرده است.

Title: روایی و پایایی نسخه فارسی شاخص راه‌رفتن پویا در مردان سالمند

http://salmandj.uswr.ac.ir/article-1-599-fa.html

اهداف: پژوهش حاضر در قالب پایان نامه کارشناسی ارشد با هدف بررسی روایی و پایایی «شاخص راه رفتن پویا» در مردان سالمند انجام شد.  مواد و روش‌ها: پژوهش علّی-مقایسه ای حاضر با 180 آزمودنی سالمند از بین کلیه سالمندان اراک در دامنه سنی 60 سال و بیشتر انجام شد. آزمودنی ها به صورت تصادفی انتخاب و معیار ورود به مطالعه تأییدیه پزشک عمومی بود. داده ها با استفاده از آزمون های تحلیل عامل اکتشافی، تحلیل مؤلفه های اصلی با چرخش واریماکس، ضریب همبستگی اسپیرمن، آلفای کرونباخ و یومن-ویتنی ارزیابی شدند.  یافته‌ها: نتایج تحقیق نشان داد که نسخۀ فارسی شاخص را هرفتن پویا در سالمندان از یک عامل تشکیل شده است. ارزش ویژه این عامل برابر با 6/091 بود که 76/133درصد واریانس را پیش‌بینی می‌کند. میزان آلفای کرونباخ در آزمون 0/953 محاسبه شد. پایایی بین ارزیاب‌ها نشان می‌دهد که همبستگی بین نمره‌های دو بار جمع آوری شده توسط دو آزمونگر، بالا و معنی‌دار بود. همچنین همبستگی آزمون مجدد، بالا و معنی‌داری بود.  نتیجه‌گیری: نتایج تحقیق نشان داد نسخه فارسی شاخص راه رفتن پویا در مردان سالمند نیز در حد قابل قبولی هماهنگ با نسخه انگلیسی آن بوده و می‌توان از آن در محیط‌های بالینی، درمانی و پژوهشی، برای شناسایی سالمندان در خطر زمین‌خوردن استفاده کرد.

Title: تأثیر دو شیوه تمرین هوازی بر تعادل ایستا و پویای سالمندان

http://salmandj.uswr.ac.ir/article-1-600-fa.html

اهداف: هدف از مطالعه حاضر، تعیین تأثیر دو شیوه تمرین هوازی بر تعادل ایستا و پویای سالمندان مرد است.  مواد و روش‌ها: پژوهش علّی-مقایسه ای حاضر با 180 آزمودنی سالمند از بین کلیه سالمندان اراک در دامنه سنی شصت سال و بیشتر انجام شد. آزمودنی‌ها به صورت تصادفی انتخاب و معیار ورود به مطالعه تأییدیه پزشک عمومی بود. داده‌ها با استفاده از آزمون‌های تحلیل عامل اکتشافی، تحلیل مؤلفه‌های اصلی با چرخش واریماکس، ضریب همبستگی اسپیرمن، آلفای کرونباخ و یو من-ویتنی ارزیابی شدند.  یافته‌ها: نتایج تحقیق نشان داد که نسخۀ فارسی شاخص را هرفتن پویا در سالمندان از یک عامل تشکیل شده است. ارزش ویژه این عامل برابر با 6/091 بود که 76/133درصد واریانس را پیش‌بینی می‌کند. میزان آلفای کرونباخ در آزمون 0/953 محاسبه شد. پایایی بین ارزیاب‌ها نشان می‌دهد که همبستگی بین نمره‌های دوبار جمع‌آوری شده توسط دو آزمونگر، بالا و معنی‌دار بود. همچنین همبستگی آزمون مجدد، بالا و معنی‌داری بود.  نتیجه‌گیری: با توجه به نتایج تحقیق پیشنهاد می‌شود که مربیان و سازمان‌ها و نهادهایی که با سالمندان سروکار دارند، از تمرین‌های دوچرخه‌سواری و پیاده‌روی و به خصوص تمرین‌های پیاده‌روی و دویدن برای کاهش به خطر افتادن این افراد استفاده کنند.

Title: پاسخ سطح گرلین آسیل‌دار پلاسمایی به تعداد گام روزانه زنان سالمند یائسه فعال و کم‌تحرک

http://salmandj.uswr.ac.ir/article-1-611-fa.html

اهداف: گرلین تام پلاسمایی در ایزوفورم های آسیل دار و بدون آسیل هنگام چاقی، هایپرگلیسمی، سوء تغذیه، ورزش و محدودیت کالری تغییر می‌کند. مطالعات علمی نیمرخ تغییرات و پاسخ هورمون گرلین تام را به دنبال ورزش‌های هوازی ناهمگون نشان داده است. ازآنجا که فعالیت پیاده‌روی به صورت آرام یا نسبتاً تند، در متن فعالیت‌های روزمره اقشار میانسال و سالمند جریان دارد، هدف پژوهش حاضر بررسی این پرسش است که آیا میانگین تعداد گام روزانه با سطح پایه ایزوفورم گرلین آسیل دار زنان یائسه به سبک‌های زندگی متفاوت، وابسته است؟ مواد و روش‌ها: چهل زن یائسه سالمند (سن 4/6±55/9 سال) بدون عوراض قلبی-عروقی و متابولیک یا ارتوپدی و بر مبنای تعداد گام روزانه به دو گروه فعّال (بیست نفر) و کم متحرک (بیست نفر) تفکیک شدند. یافته‌ها: میانگین تعداد گام روزانه گروه فعّال (2659±8022) نسبت به گروه کم تحرک (913±3450) به صورت معنادار بالاتر بود. غلظت‌های پایه گرلین فعّال در دو گروه فعّال (195.4±131 pg/ml) و کم تحرک (73±pg/ml 165/4) تفاوت معناداری نداشت همچنین ارتباط معناداری میان سطح گرلین آسیل دار و تعداد گام روزانه آنان به دست نیامد (05/P<0). تغییرات انسولین ناشتا در دو گروه تفاوت معناداری نداشت و ارتباط آن با تعداد گام روزانه نیز معنادار نبود (05/P<0). با این حال پارامترهای آنتروپومتری BMI, % BF, WHR در دو گروه، معنادار و ارتباط معکوس با تعداد گام روزانه را نشان داد (0/05>P). نتیجه‌گیری: افزایش حجم فعالیت بدنی زنان سالمند یائسه با کاهش عوامل خطرزای بیماری‌های مزمن و کنترل چاقی ارتباط پیدا می‌کند. به علاوه افزایش تعداد گام روزانه (حداقل به اندازه 8000 گام در روز) تاثیری بر تغییر سطوح هورمون‌های گرلین آسیل دار و انسولین ناشتا نداشت. به نظر می‌رسد که مطابق شواهد علمی آستانه حداقل 10000 گام روزانه، احتمالاً برای دستیابی به ایجاد پاسخ سازگاری‌های متابولیک این هورمون اشتها در افراد بزرگسال و میانسال قابل توجه باشد که به مراتب پایین‌تر از حجم فعالیت بدنی زنان فعّال در این مطالعه مقطعی است.

Title: پیش‌بینی کارکردهای خاطره‌گویی براساس مدل پنج عاملی شخصیت در سالمندان

http://salmandj.uswr.ac.ir/article-1-631-fa.html

اهداف: هدف از پژوهش حاضر بررسی پیش بینی کارکردهای خاطره‌گویی براساس مدل پنج عاملی شخصیت در سالمندان است. مواد و روش‌ها: تعداد 242 فرد سالمند بالای شصت سال عضو کانون‌های بازنشستگی شهر شیراز به روش نمونه‌گیری در دسترس، انتخاب شدند. افراد نمونه با تکمیل مقیاس کارکردهای خاطره‌گویی و پرسش‌نامه پنج عامل بزرگ شخصیت گلدبرگ در این پژوهش شرکت کردند. از میان افراد گروه نمونه چهل نفر به دلیل عدم تکمیل کامل پرسش‌نامه‌ها از پژوهش حذف شدند. شرکت تمامی آزمودنی‌ها با رضایت آگاهانه بوده است. برای تجزیه و تحلیل داده‌ها در بخش آمار توصیفی، از شاخص‌های میانگین، انحراف استاندارد و ضریب همبستگی پیرسون و در بخش آمار استنباطی از رگرسیون چند گانه همزمان برای پیش‌بینی کارکردهای خاطره‌گویی استفاده شده است. در اجرای عملیات تجزیه و تحلیل داده‌ها از نرم‌افزار آماری SPSS 16 استفاده گردید.  یافته‌ها: نتایج تحلیل رگرسیون چندگانه هم زمان نشان داد که عامل روان نژندگرایی مدل پنج عاملی شخصیت، کارکردهای خاطره‌گویی حفظ صمیمیت (25/P≤0/001, β=0) و تجدید خاطرات تلخ (28/P≤0/001, β=0) را پیش‌بینی کرد. همچنین عامل برون‌گرایی مدل پنج عاملی شخصیت کارکرد خاطره‌گویی آموزش/آگاهی را پیش‌بینی نمود (18/P<0/05, β=0). نتیجه‌گیری: در تبیین یافته‌های پژوهش حاضر می‌توان این گونه نتیجه‌گیری کرد که افراد، دچار میزان بالای آسیب‌پذیری هیجانی به عنوان یک صفت، به منظور نشخوار خاطرات تلخ و زنده نگه داشتن یاد دوستانی که دیگر در بین آن‌ها نیستند خاطرات خود را مرور یا بازگو می‌کنند و افراد برون‌گرا با هدف آموزش درس زندگی و انتقال تجربیات خود به دیگران از خاطره‌گویی استفاده می‌کنند. به درمانگران پیشنهاد می‌شود که در درمان خاطره‌گویی، بر کارکردهای سازگارانه‌تر خاطره گویی (مانند، هویت و حل مسئله، گفتگو و آموزش/ آگاهی) تاکید نمایند و این گونه کارکردها را آموزش دهند.

Title: روایی و پایایی نسخه فارسی‌شده مقیاس فعالیت‌های کارساز روزمره زندگی لوتون در مبتلایان به دمانس

http://salmandj.uswr.ac.ir/article-1-638-fa.html

اهداف: مقیاس فعالیت‌های کارساز روزمره زندگی لوتون یکی از رایج‌ترین ابزارهای ارزیابی فعالیت‌های کارساز زندگی در افراد مبتلا به دمانس محسوب می‌شود، اما تاکنون روایی و پایایی آن در جامعه ایرانی صورت نگرفته است. هدف این مطالعه، بررسی روایی و پایایی نسخه فارسی شده مقیاس فعالیت های کارساز روزمره زندگی لوتون در بیماران مبتلا به دمانس بود.  مواد و روش‌ها: به منظور ترجمه پرسش‌نامه، از الگوی رویکرد ارزیابی بین المللی کیفیت زندگی (IQOLA) استفاده گردید. پس از انجام ترجمه رو به جلو و برگردان به منظور تعیین روایی، نسخه ترجمه شده لوتون که شامل هشت مورد از جمله توانایی استفاده از تلفن، خرید، آماده کردن غذا، خانه‌داری، شستن لباس‌ها، نوع حمل ونقل، مصرف داروها و انجام امور مالی است، در اختیار پنج نفر کارشناس خبره در این حوزه قرار گرفت. برای بررسی پایایی آزمون نیز از شیوه آزمون-آزمون مجدد پس از یک هفته و پایایی بین آزمونگران استفاده شد. همسانی درونی آیتم‌ها نیز با ضریب آلفای کرونباخ مورد بررسی قرار گرفت. نتایج براساس اطلاعات حاصل از شصت فرد سالمند مبتلا به دمانس مراجعه‌کننده به انجمن آلزایمر ایران گزارش گردیده است. یافته‌ها: توافق نظر کارشناسان در مورد روایی محتوایی بدین صورت بود که مناسب بودن پنج سؤال را خوب و بالاتر ارزیابی کردند، مناسب بودن یک سؤال را خیلی خوب و دو سؤال را از متوسط به بالا ارزیابی کردند. در تمامی موارد، در توزیع ارزیابی داوران از سؤالات تفاوت آماری معناداری مشاهده نشد (05/P>0). همبستگی کل آزمون در اجرای مرتبه اول و دوم (993/r=0 و SEM=0/238) و (0/996- 988/CI:0) و همبستگی کل بین آزمونگر اول و دوم بسیاربالا بود (001/r=0/961 ،P<0) که حاکی از پایایی خوب آزمون است. همبستگی سؤالات با نمره کل آزمون متوسط .(0.606>r>0.427) نتیجه‌گیری: نتایج نشان می‌دهد که نسخه فارسی مقیاس لوتون از روایی مطلوبی برخوردار است. همچنین میزان ضریب همبستگی آزمون در اجراهای متوالی و اجرا توسط آزمونگران متفاوت بالا بود که خود حاکی از اعتبار بالای آزمون است. این پرسش‌نامه به عنوان یک ابزار مناسب در ارزیابی فعالیت‌های کارساز روزمره زندگی در بیماران مبتلا به دمانس است و می‌توان آن را در اختیار پژوهشگران، متخصصان بالینی و مراکز درمانی قرار داد تا در کنار دیگر آزمون‌ها، ارزیابی دقیق تر و جامع‌تری از این بیماران به عمل آید.
